# Clobetasol promotes neuromuscular plasticity in mice after motoneuronal loss via sonic hedgehog signaling, immunomodulation and metabolic rebalancing

**DOI:** 10.1038/s41419-021-03907-1

**Published:** 2021-06-16

**Authors:** Nunzio Vicario, Federica M. Spitale, Daniele Tibullo, Cesarina Giallongo, Angela M. Amorini, Grazia Scandura, Graziana Spoto, Miriam W. Saab, Simona D’Aprile, Cristiana Alberghina, Renata Mangione, Joshua D. Bernstock, Cirino Botta, Massimo Gulisano, Emanuele Buratti, Giampiero Leanza, Robert Zorec, Michele Vecchio, Michelino Di Rosa, Giovanni Li Volti, Giuseppe Lazzarino, Rosalba Parenti, Rosario Gulino

**Affiliations:** 1 Department of Biomedical and Biotechnological Sciences, Section of Physiology, University of Catania, 95123 Catania, Italy; 2 Molecular Preclinical and Translational Imaging Research Centre - IMPRonTE, University of Catania, 95125 Catania, Italy; 3 Department of Biomedical and Biotechnological Sciences, Section of Biochemistry, University of Catania, 95123 Catania, Italy; 4 Department of Medical, Surgical Sciences and Advanced Technologies G.F. Ingrassia, University of Catania, 95123 Catania, Italy; 5 Department of Basic Biotechnological Sciences, Intensive and Perioperative Clinics, Catholic University of Rome, 00168 Rome, Italy; 6 Department of Neurosurgery, Brigham and Women’s Hospital, Harvard University, Boston, MA 02155 USA; 7 Hematology Unit, Annunziata Hospital, 87100 Cosenza, Italy; 8 Department of Drug and Health Sciences, University of Catania, 95123 Catania, Italy; 9 International Centre for Genetic Engineering and Biotechnology (ICGEB), 34149 Trieste, Italy; 10 Laboratory of Cell Engineering, Celica Biomedical, 1000 Ljubljana, Slovenia; 11 Laboratory of Neuroendocrinology – Molecular Cell Physiology, Institute of Pathophysiology, Faculty of Medicine, University of Ljubljana, 1000 Ljubljana, Slovenia; 12 Rehabilitation Unit, AOU Policlinico G. Rodolico, 95123 Catania, Italy; 13 Department of Biomedical and Biotechnological Sciences,Section of Pharmacology, University of Catania, 95123 Catania, Italy; 14 Department of Biomedical and Biotechnological Sciences, Section of Anatomy, Histology and Movement Sciences, University of Catania, 95123 Catania, Italy

**Keywords:** Biochemistry, Diseases of the nervous system, Glial biology, Physiology

## Abstract

Motoneuronal loss is the main feature of amyotrophic lateral sclerosis, although pathogenesis is extremely complex involving both neural and muscle cells. In order to translationally engage the sonic hedgehog pathway, which is a promising target for neural regeneration, recent studies have reported on the neuroprotective effects of clobetasol, an FDA-approved glucocorticoid, able to activate this pathway via smoothened. Herein we sought to examine functional, cellular, and metabolic effects of clobetasol in a neurotoxic mouse model of spinal motoneuronal loss. We found that clobetasol reduces muscle denervation and motor impairments in part by restoring sonic hedgehog signaling and supporting spinal plasticity. These effects were coupled with reduced pro-inflammatory microglia and reactive astrogliosis, reduced muscle atrophy, and support of mitochondrial integrity and metabolism. Our results suggest that clobetasol stimulates a series of compensatory processes and therefore represents a translational approach for intractable denervating and neurodegenerative disorders.

## Introduction

Amyotrophic lateral sclerosis (ALS) is a progressive neurodegenerative disease that affects both upper and lower motoneurons (MNs) [1]. While relatively rare with an incidence of ~2 per 100,000, the clinical course is severe and prognosis dismal with the vast majority of patients dying from respiratory failure 2–5 years after diagnosis [2]. Despite extensive basic and clinical research efforts, the causes of the disease remain to be fully elucidated and no truly effective therapies are yet available [3, 4]. Therefore, the use of animal models appropriately designed to recapitulate the pathology represents a valuable tool for defining new therapeutic approaches [5].

Several data on the pathogenesis of ALS have defined a focal origin in the central nervous system (CNS), where multiple factors contribute to creating a toxic milieu [6, 7], but an active role of both muscle cells and axon terminals in causing retrograde degeneration of MNs has been also proposed as a triggering mechanism [8–10]. Consistently, during the early stages of ALS progression, muscle undergoes denervation, and the gold standard for ALS diagnosis remains based on nerve conduction analysis and electromyography (EMG) [11]. In our view, the use of reductionist in vivo models would help in the step-by-step dissection of ALS pathogenesis. To this regard, the focal removal of confined populations of spinal MNs by injection of cholera toxin-B conjugated to saporin (CTB-Sap), has proven to be useful in mimicking respiratory dysfunction [12], dysphagia [13], and focal MN loss [14, 15]. It is also worth noting that although MN loss is certainly a critical hallmark of ALS, glial and muscle cells are also involved [16, 17]. Neuroinflammation in ALS has been reported to disrupt homeostatic neuroglial interactions [1]. Moreover, the onset of the disease is known to begin after degeneration is already severe, because plastic mechanisms can compensate for the initial MN loss [18].

Several studies reported that sonic hedgehog (SHH) may represent a crucial regulator of neuroinflammation, neuroprotection, and plasticity [19–22]. SHH serves as a morphogen controlling neural tube patterning during development [23], and also self-renewal and differentiation of neural precursor cells within the postnatal brain [24]. The secreted SHH ligand binds to its receptor Patched, thereby activating smoothened (SMO); this ultimately leads to the nuclear translocation of transcription factors Gli1–3, inducing the expression of target genes [25]. Interestingly, SHH signaling has been shown to play a role in ALS pathogenesis. In particular, the reduction of Gli1 has been reported within the MNs of SOD1^G93A^ mice and SHH has been shown to be cytoprotective in vitro [20]. Accordingly, cerebrospinal fluid collected from ALS patients may actually inhibit SHH signaling in vitro [26]. Therefore, SHH signaling represents a druggable target in neurodegenerative disorders [27]. In particular, small molecules targeting SMO have recently been explored in cancer (i.e., SMO antagonists), stroke, and demyelinating disorders (i.e. SMO agonists) [28, 29]. The glucocorticoid clobetasol, which also acts as a SMO agonist, has received attention as a potential therapeutic agent capable of remyelination and neuroprotection/repair [30–32]. However, few data are available regarding the potential effects of SMO activation in MN disease. Therefore, herein we sought to examine the effects of clobetasol treatment in a murine CTB-Sap model of MN degeneration.

## Results

### Clobetasol promotes behavioral improvement by inducing muscle reinnervation

**Fig. 1:**
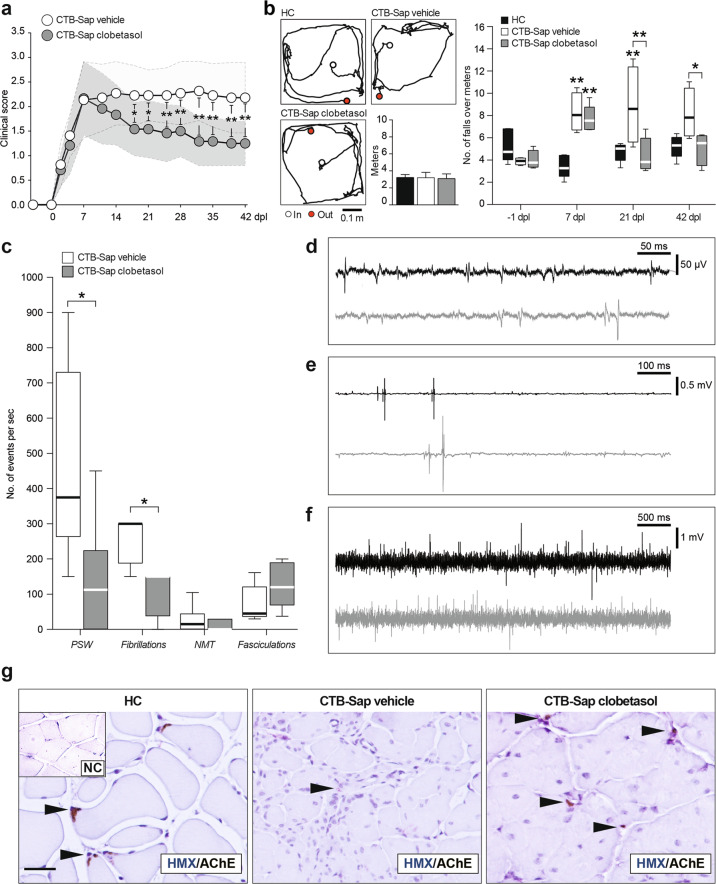
Clobetasol ameliorates behavioral impairment of CTB-Sap-induced MN ablation. **a** Repeated measures of a clinical score of motor impairment during the time-course of CTB-Sap-induced motoneuronal depletion in CTB-Sap vehicle and CTB-Sap clobetasol groups; data are shown as mean ± SEM of *n* = 12 mice per group; *p-value < 0.05 and **p-value < 0.01 between groups; two-way repeated-measures ANOVA. **b** Representative tracks of HC, CTB-Sap vehicle, and CTB-Sap clobetasol groups and quantification of the distance (expressed as mean meters ± SEM of *n* = 5 mice per time-point, as repeated measures) covered during 2 min performance on open-field grid walk test and quantification of the number of footfalls over a meter at −1, 7, 21 and 41 dpl in HC, CTB-Sap vehicle, and CTB-Sap clobetasol groups; data are shown as standard box-and-whiskers of *n* = 5 mice per group; *p-value < 0.05 and **p-value < 0.01 versus HC and between groups; two-way repeated-measures ANOVA. **c**–**f** Quantification and representative electromyographic profile of positive sharp waves (PSW, **c** and **d**), fibrillations (**c** and **d**), pseudo-neuromyotonia (NMT, **c** and **e**), and fasciculations (**c** and **f**) observed in denervated GM in CTB-Sap vehicle (black tracks in **d**–**f**) and CTB-Sap clobetasol groups (gray tracks in **d**–**f**); data are expressed as a number of events per second and showed as standard box-and-whiskers of *n* = 4 mice per group; measures were taken from distinct samples; *p-value < 0.05 versus CTB-Sap vehicle; two-sided Student’s *t*-test. **g** Representative pictures of AChE immunostaining and hematoxylin (HMX) and negative control (NC) on left GM of HC, CTB-Sap vehicle, and CTB-Sap clobetasol; scale bar: 25 μm. Dpl: days post lesion.

We induced a spinal MN depletion by injecting CTB-Sap toxin into the left gastrocnemius muscle (GM) and assessed the impact of clobetasol treatment on postural and motor performance, as compared to vehicle-treated CTB-Sap-injected mice. Clinical score assessment showed that all animals had a rapid worsening of the left hindlimb postural and motor ability during the first week after lesion (Fig. 1a). Then, clobetasol-treated animals started to ameliorate their clinical score at 14–21 days post lesion (dpl), and this condition was retained up to 42 dpl, whereas vehicle-treated lesioned animals did not ameliorate spontaneously (Fig. 1a). An open field grid walk test was performed in order to evaluate the motor coordination, finding that MN ablation did not affect the distance covered by mice during the grid walk performance (Fig. 1b), but it significantly increased the number of footfalls at 7 dpl as compared to healthy control (HC) group (Fig. 1b). Notably, clobetasol reverted this impairment, promoting a significant reduction of the number of footfalls at 21 and 42 dpl, as compared to vehicle-treated lesioned mice (Fig. 1b).

EMG recordings from the left GM showed typical clinical evidence of denervation, such as positive sharp waves (PSW), fibrillation (Fig. 1c, d), pseudo-neuromyotonia (NMT, Fig. 1c–e), and fasciculations (Fig. 1c–f). Notably, clobetasol-treated CTB-Sap-lesioned mice showed a significant 70% reduction of PSW and fibrillation compared to the vehicle-treated group (Fig. 1c). This evidence was coupled with a reduced number and size of AChE-positive spots in the left GM and a near-normal pattern in clobetasol-treated mice (Fig. 1g). Together, these data suggest that clobetasol exerts restorative behavioral effects and promotes muscle reinnervation after neurotoxic MN removal.

### Clobetasol promotes spinal cord plasticity acting on SHH signaling pathway

**Fig. 2:**
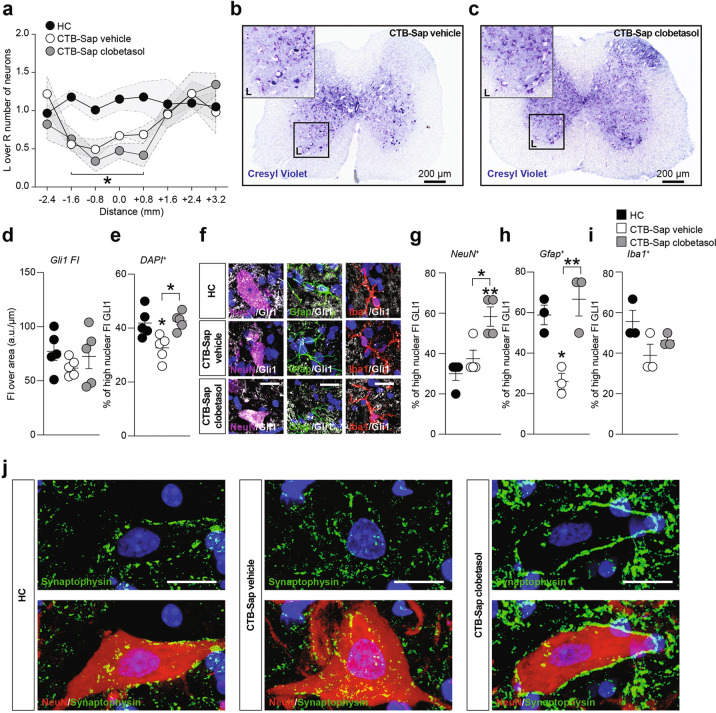
Clobetasol modulates Gli1 nuclear translocation and plasticity on MN-depleted spinal cord. **a**–**c** Quantification of the number of motoneurons in left (L) and right (R) Rexed lamina IX in HC, CTB-Sap vehicle and CTB-Sap clobetasol groups (**a**) and representative pictures of spinal cord sections stained with cresyl violet of CTB-Sap vehicle (**b**) and CTB-Sap clobetasol (**c**); data in (**a**) are expressed as the ratio of L over R motoneurons number ± SEM (shade) of *n* = 8 sections of *n* = 5 mice per group; *p-value < 0.05 versus HC; one-way ANOVA; scale bar in (**b**) and (**c**): 200 μm. **d** and **e** Quantification of the total Gli1 fluorescence intensity (FI) over μm (**d**) and of the percentage of high nuclear FI of Gli1 (**e**) in HC, CTB-Sap vehicle, and CTB-Sap clobetasol spinal cord cell population; data are shown as scatter dot plots and mean ± SEM of *n* ≥ 3 mice per group; *p-value < 0.05 versus HC and between groups; one-way ANOVA. **f**–**i** Representative pictures and quantification of the proportion of NeuN positive (**f** and **g**), Gfap positive (**f** and **h**), and Iba1 positive (**f** and **i**) cells with high nuclear FI of Gli1 in HC, CTB-Sap vehicle, and CTB-Sap clobetasol spinal cord; data are shown as scatter dot plots and mean ± SEM of *n* ≥ 3 mice per group; *p-value < 0.05 and **p-value < 0.01 versus HC and between groups; one-way ANOVA. **j** Representative pictures of NeuN and Synaptophysin immunofluorescence on spinal cord motoneurons of HC, CTB-Sap vehicle, and CTB-Sap clobetasol; scale bar: 20 μm.

Neuropathological assessment of lumbar spinal cord sections revealed a depletion of about 50% of MNs in the lesioned (i.e. left) side compared to the intact spinal cord side (Fig. 2a–c), and this rate of MN loss was present in all CTB-Sap-lesioned mice, regardless of the drug treatments. This suggests that the functional amelioration and muscle reinnervation is linked to compensatory plastic changes within the spared MN population.

In order to link our observations with a potential activation of the SHH signaling pathway in the spinal cord, we analyzed Gli1 nuclear translocation in resident cell populations of HC and CTB-Sap mice treated with either vehicle or clobetasol. Results showed that the total fluorescence intensity (FI) of Gli1 was unchanged (Fig. 2d), whereas a significant reduction of nuclear Gli1 FI was found in CTB-Sap-lesioned mice at 42 dpl, but near-normal levels were found in CTB-Sap mice treated with clobetasol (Fig. 2e). To analyze such a modulation into specific cell populations, we evaluated the Gli1 nuclear translocation in NeuN (i.e. neurons), Gfap (i.e. astrocytes), and Iba1 (i.e. microglia) positive cells. Our data highlighted that clobetasol was able to significantly increase the amount of nuclear Gli1 in NeuN-positive cells by about 2 folds (Fig. 2f, g). Moreover, a reduction of nuclear Gli1 was found in the astroglial cell population after lesion but, importantly, near-normal levels were present in clobetasol-treated mice (Fig. 2f–h). Finally, neither CTB-sap lesion nor clobetasol treatment did affect nuclear translocation of Gli1 in microglia (Fig. 2f–i). We also analyzed the potential effect of clobetasol in modulating spinal MN plasticity. Lesioned animals treated with clobetasol showed a remarkable increase of newly formed synaptic contacts labeled with synaptophysin, as compared to both vehicle-treated lesioned mice and HC (Fig. 2j).

### Clobetasol modulates metabolic changes in MN-depleted spinal cord

**Fig. 3:**
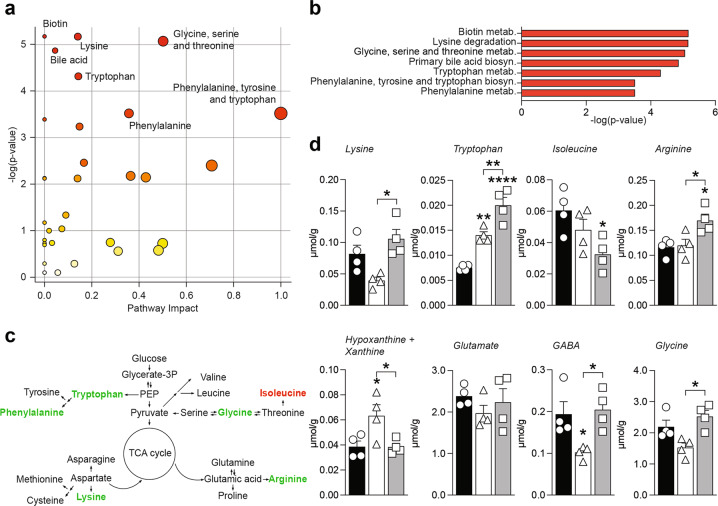
Clobetasol modifies spinal cord metabolism and relevant neurotransmitters. **a** and **b** Summary plot for pathway analysis (**a**) and quantitative enrichment analysis (**b**) for metabolites levels in CTB-Sap vehicle versus CTB-Sap clobetasol; **c** Schematic representation of amino acids biosynthesis showing significantly upregulated (green) and downregulated (red) metabolites in CTB-Sap clobetasol versus CTB-Sap vehicle group. **d** Metabolites levels in the spinal cord of HC, CTB-Sap vehicle and CTB-Sap clobetasol mice; data are shown as mean ± SEM and scattered dot plots of *n* = 4 mice per group; *p-value < 0.05, **p-value < 0.01, ****p-value < 0.0001 versus HC or between groups; one-way ANOVA.

We examined the metabolic profiles of the intact and lesioned spinal cord either with or without clobetasol treatment (Figs. S1 and S2). Our results suggest that amino acids biosynthesis metabolic pathways (Fig. 3a–c), lysine, tryptophan, and arginine levels were significantly modulated in CTB-Sap clobetasol versus CTB-Sap vehicle mice (Fig. 3d). We also observed that isoleucine levels were slightly, but not significantly, reduced in CTB-Sap vehicle group and significantly decreased in CTB-Sap clobetasol mice (Fig. 3d). Among the highest contributors to the PCA analysis (Fig. S2) we found that hypoxanthine and xanthine were of importance and they were significantly higher in CTB-Sap vehicle mice (Fig. 3d), while clobetasol reverted such accumulation to the level of healthy spinal tissue (Fig. 3d). Results also revealed that CTB-Sap lesion was associated with a reduction of GABA and glycine, both involved in spinal inhibitory circuitry and that clobetasol was able to restore near-normal levels of both neurotransmitters (Fig. 3d). Interestingly, even in presence of a reduced number of MNs in the lesioned spinal cord side (about 50%), no significant changes were observed in glutamate levels (Fig. 3d), thus suggesting that spared MNs could probably receive increased glutamate signaling as compared to HC MNs.

### Clobetasol reduces pro-inflammatory resident cells in MN-depleted spinal cord

**Fig. 4:**
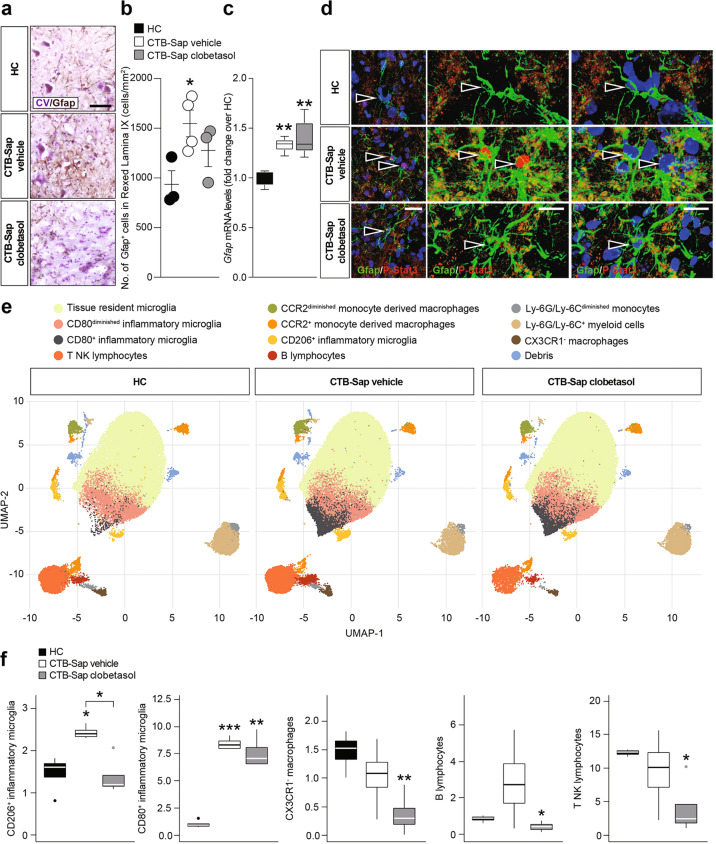
Clobetasol-mediated reduction of pro-inflammatory cells in MN-depleted spinal cord. **a** and **b** Representative pictures of Gfap immunostaining (**a**) and quantification of the number of positive cells for Gfap (**b**) in HC, CTB-Sap vehicle, and CTB-Sap clobetasol spinal cord; data are shown as scatter dot plot and mean ± SEM of *n* ≥ 3 mice per group; *p-value < 0.05 versus HC; one-way ANOVA; scale bar in (**a**): 25 μm. **c** mRNA levels of Gfap in the spinal cord of in HC, CTB-Sap vehicle, and CTB-Sap clobetasol spinal cord; data are shown as standard box-and-whiskers plot and mean ± SEM of *n* ≥ 3 mice per group; **p-value < 0.01 versus HC; one-way ANOVA. **d** Representative pictures and confocal deconvolutions of Gfap and P-Stat3 staining in HC, CTB-Sap vehicle, and CTB-Sap clobetasol spinal cord; arrowheads indicate astrocytes nuclei and in CTB-Sap vehicle P-Stat3 positive nuclei; scale bar: 20 μm. **e** Uniform manifold approximation and projection (UMAP) representation of the populations identified through flow-cytometry in the three different conditions. **f** Standard box-and-whiskers plot representing the five subpopulations that presented a significant modulation between HC, CTB-Sap vehicle, and CTB-Sap clobetasol mice. Plots show data from *n* = 4 mice per group; *p-value < 0.05 versus CTB-Sap vehicle; one-way ANOVA.

MN ablation was associated with an increased total number of Gfap^+^ astrocytes in the Rexed lamina IX of CTB-Sap-lesioned mice treated with vehicle, whereas clobetasol-treated group showed an apparent but not statistically significant increase over control levels (Fig. 4a, b). However, the total *Gfap* mRNA levels measured in the spinal cord were similar in both lesioned groups, with a significant increase above control levels (Fig. 4c). In order to clarify whether spinal astrocytes were polarized towards a reactive state, we analyzed phospho-stat-3 (P-Stat3) expression in spinal Gfap^+^ cells (Fig. 4d). Our evidence supports the hypothesis that MN depletion increases the number of reactive astrocytes, and that clobetasol was able to reduce nuclear P-Stat3 expression in lamina IX, thus partially reversing the effects of the lesion.

We also performed a multidimensional flow cytometry analysis of spinal cord samples to characterize the myeloid compartment (i.e. tissue-resident microglia phenotypes, monocytes, monocytes-derived macrophages) and, due to shared markers, also B and non-B (i.e. T NK) lymphocytes (Fig. S3). Interestingly, our results showed that MN depletion increased the frequency of CD206^+^ and CD80^+^ inflammatory microglia and that clobetasol, besides reducing polarized microglial cells (Fig. 4e, f), also reduced the total amount of B and T NK lymphocytes (Fig. 4e, f). These findings suggest that clobetasol was able to attenuate the pro-inflammatory signaling in the spinal cord, thus likely promoting plasticity and compensatory processes.

### Clobetasol promotes trophic processes in the denervated muscle

**Fig. 5:**
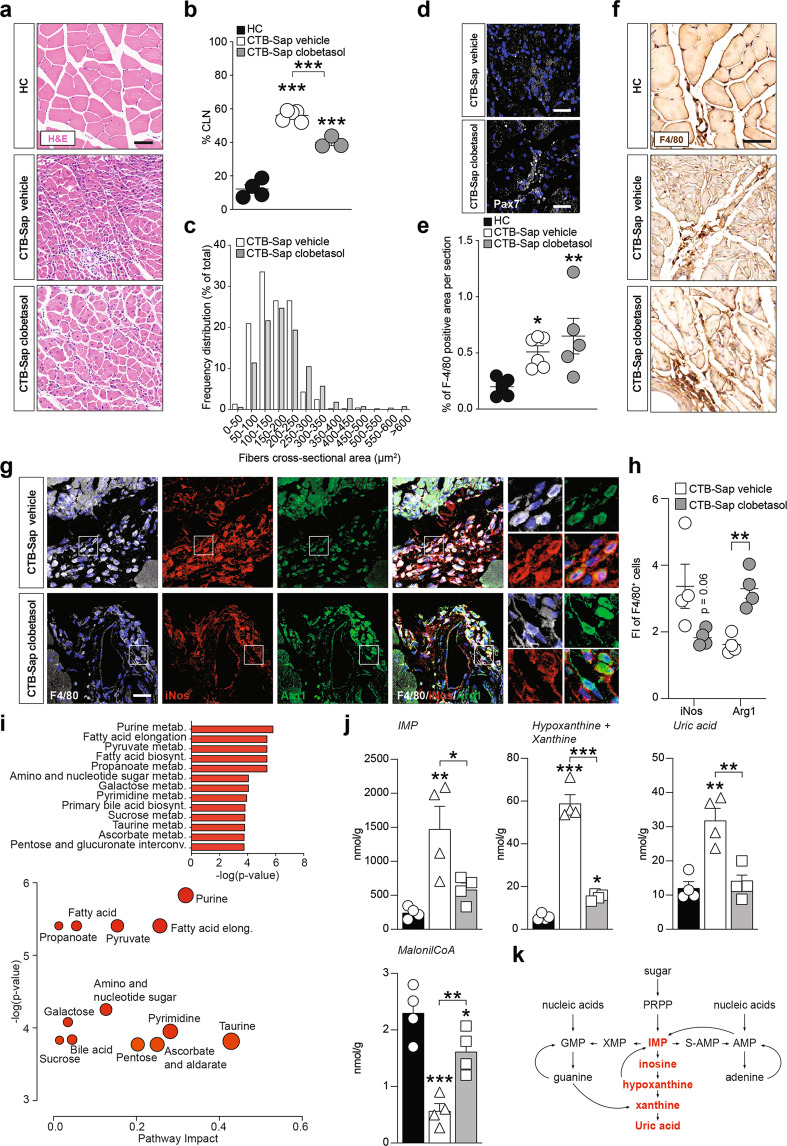
Clobetasol increases muscle fiber size and metabolism. **a** Representative pictures of H&E staining in left GM of HC, CTB-Sap vehicle, and CTB-Sap clobetasol mice; scale bar: 25 μm. **b** Quantification of the percentage of centrally located nuclei (CLN) in HC, CTB-Sap, and CTB-Sap clobetasol-treated mice; data are % of CLN over total muscle fibers and are shown as scatter dot plot and mean ± SEM of *n* ≥ 3 mice per group; ***p-value < 0.001 versus HC or between groups; one-way ANOVA. **c** Frequency plot of the mean fiber cross-sectional area expressed in μm^2^ in the left GM of CTB-Sap vehicle and CTB-Sap clobetasol mice. **d** Representative pictures of Pax7 positive cells in the left GM of CTB-Sap vehicle and CTB-Sap clobetasol mice. **e** and **f** Quantification of the percentage of F4/80 positive area per section (**e**) and representative pictures of F4/80 immunostaining (**f**) in the left GM of HC, CTB-Sap vehicle, and CTB-Sap clobetasol mice; data in (**e**) are shown as scatter dot plot and mean ± SEM of *n* ≥ 3 mice per group; *p-value < 0.05 and **p-value < 0.01 versus HC; one-way ANOVA; scale bar in (**f**): 25 μm. **g** and **h** Representative pictures of F4/80, iNos and Arg1 immunostaining (**g**) and quantification of the FI of iNOS and Arg1 in F4/80 positive cells (**h**) in the left GM of CTB-Sap vehicle and CTB-Sap clobetasol mice; data in (**h**) are shown as scatter dot plot and mean ± SEM of *n* ≥ 3 mice per group; **p-value < 0.01 versus CTB-Sap vehicle; two-sided Student’s *t*-test. Scale bar in (**g**): 25 μm. **i** Summary plot for quantitative enrichment analysis and pathway analysis for metabolites levels in CTB-Sap vehicle versus CTB-Sap clobetasol; **j** Metabolites levels in the left GMs of HC, CTB-Sap vehicle, and CTB-Sap clobetasol mice; data are shown as mean ± SEM and scattered dots plot of *n* = 4 mice per group; *p-value < 0.05 and **p-value < 0.01 versus HC or between groups; one-way ANOVA. **k** Schematic representation of purine metabolism showing significantly upregulated metabolites in red in CTB-Sap vehicle versus CTB-Sap clobetasol group.

In order to associate the CNS effects induced by clobetasol with the observed functional restoration, we analyzed the morphological and metabolic changes of muscles. Our data indicate that, after the partial denervation caused by MN removal, muscle fibers in GM showed an increased number of centrally located nuclei (CLN) as compared to HC (Fig. 5a, b), but clobetasol treatment significantly reduced the proportion of myofibers with CLN (Fig. 5b). To assess muscle atrophy, we evaluated the cross-sectional area of myofibers in GM of CTB-Sap mice treated with vehicle or clobetasol, finding an overall increase of fiber area in clobetasol-treated mice compared to vehicle-treated, even if it did not reach the physiological pattern found in HC mice (Fig. 5a–c). Such an amelioration was coupled with increased Pax7^+^ cells in GM of clobetasol-treated mice (Fig. 5d). Furthermore, we found a significantly increased amount of F4/80^+^ infiltrating macrophages in both vehicle-treated and clobetasol-treated denervated muscles (Fig. 5e, f). To determine the phenotype of infiltrating macrophages in GM of vehicle-treated and clobetasol-treated mice, we analyzed F4/80^+^ cell phenotype (Fig. 5g) by quantifying those highly expressing iNos or Arg1. The results revealed that clobetasol was able to significantly increase the proportion of anti-inflammatory infiltrating macrophages into denervated GM (Fig. 5g, h), thus likely sustaining a pro-regenerative microenvironment. Finally, we analyzed the metabolic effects induced by denervation onto GMs (Fig. S4–S6). PCA analysis revealed that inosine monophosphate (IMP), hypoxanthine, xanthine, and uric acid played a major role. This analysis also highlighted that purine nucleotide catabolism and fatty acid biosynthesis and elongation were strictly regulated in denervated GM (Fig. 5i). We observed a robust accumulation of IMP, hypoxanthine, xanthine, and uric acid in CTB-Sap vehicle group (Fig. 5j, k), which suggests an overproduction of reactive oxygen species in the muscular microenvironment, and a decreased availability of malonil-CoA for fatty acid biosynthesis (Fig. 5j). Such evidence suggests that denervation shapes purine and fatty acid metabolism in the muscle and that clobetasol was able to revert this condition (Fig. 5j), promoting favorable metabolic programs after CTB-Sap-induced muscle denervation.

### Clobetasol reverts denervation-induced mitochondrial fission and increases energy substrates

**Fig. 6:**
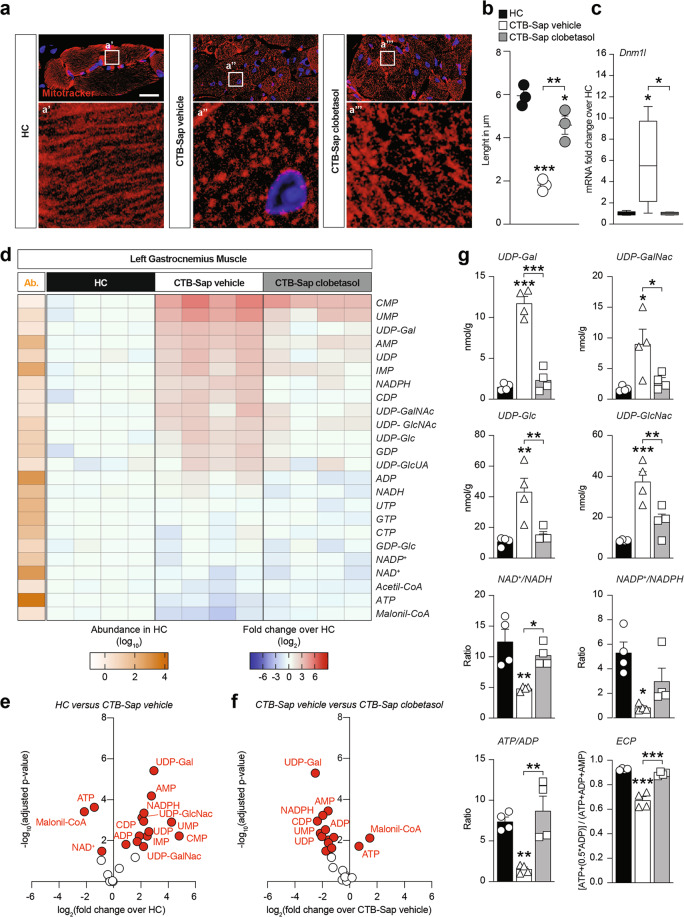
Clobetasol reverts denervation-induced mitochondrial fission and increases energy substrates. **a** and **b** Representative pictures of MitoTracker immunostaining (**a**) and quantification of the mitochondrial length expressed in μm (**b**) in the left GM of HC, CTB-Sap vehicle, and CTB-Sap clobetasol mice; data in (**b**) are shown as scatter dot plot and mean ± SEM of *n* ≥ 3 mice per group; *p-value < 0.05, **p-value < 0.01 and ***p-value < 0.001 versus HC or between groups; one-way ANOVA; scale bar in (**a**): 25 μm. **c** mRNA expression levels of *Dnm1l* in the left GM of HC, CTB-Sap vehicle and CTB-Sap clobetasol mice; data are fold changes over HC and are shown as standard box-and-whiskers plot of *n* = 5 mice per group; *p-value < 0.05 versus HC or between groups; one-way ANOVA. **d** Heat maps of 24 metabolites in the left GM HC, CTB-Sap vehicle and CTB-Sap clobetasol at 42 dpl showing the abundance (Ab.) in HC and the relative changes in CTB-Sap vehicle and CTB-Sap clobetasol as compared to HC; data are shown as log_10_ abundance and log_2_ FC over HC of *n* = 4 mice per group. **e** and **f** Volcano plots of metabolites levels in HC versus CTB-Sap vehicle (**e**) and CTB-Sap vehicle versus CTB-Sap clobetasol (**f**). **g** Metabolites levels and ratios in the left GMs of HC, CTB-Sap vehicle, and CTB-Sap clobetasol mice; data are shown as mean ± SEM and scattered dots plot of *n* = 4 mice per group; *p-value < 0.05 and **p-value < 0.01 versus HC or between groups; one-way ANOVA.

Mitochondrial morphology was assessed in muscle sections, finding that denervation strongly affected mitochondrial fitness, as shown by the significant reduction of mitochondrial length (Fig. 6a, b) and by the significant increase of dynamin-like 1 (*Dnm1l*) mRNA levels, which is an essential gene controlling mitochondrial fission (Fig. 6c). Notably, clobetasol-treated CTB-Sap-lesioned mice showed increased mitochondrial length, coupled with a normalization of *Dnm1l* mRNA levels (Fig. 6a–c), suggesting a restored mitochondrial homeostasis and energy metabolism in clobetasol-treated mice. Then, we analyzed phosphate nucleotides, nicotinic coenzymes, and glycosylated UDP-derivatives (Fig. 6d and Fig. S7). The results highlighted a significant accumulation of glycosylated UDP-derivatives and monophosphate nucleotides coupled with a reduction of ATP in GM of CTB-Sap mice treated with vehicle (Fig. 6e), and these effects were reverted by clobetasol (Fig. 6f). Indeed, UDP-Gal, UDP-GalNac, UDP-Glc, and UDP-GlcNac (Fig. 6g), were all significantly increased in CTB-Sap-vehicle mice, whereas clobetasol treatment induced their normalization (Fig. 6g).

Notably, by measuring the levels of nicotinic coenzymes, we found a strong decrease of the oxidized over reduced form of NAD and NADP, thus indicating a reduced mitochondrial redox potential in denervated GM as compared to HC and clobetasol-treated lesioned group (Fig. 6g). Finally, we evaluated the ratio between ATP and ADP and the energy charge potential (ECP). Our data highlighted an overall increase of ATP/ADP ratio and ECP induced by clobetasol versus vehicle group (Fig. 6g), thus supporting the hypothesis of restored energy metabolism.

### Human ALS spinal cord shows impaired SHH signaling pathway

**Fig. 7:**
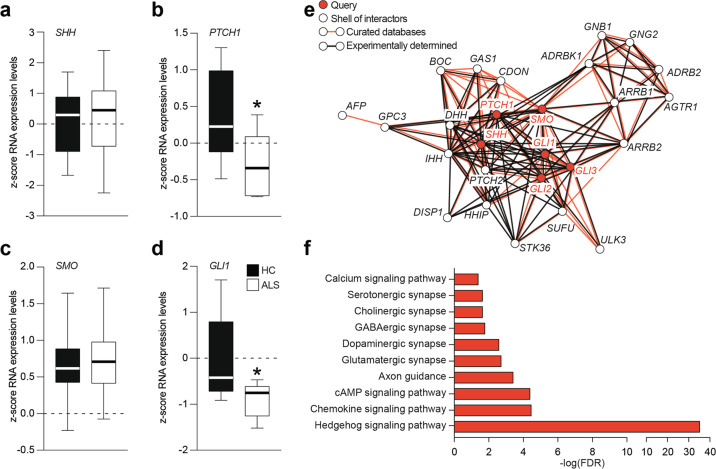
SHH signaling pathway is downregulated in ALS. **a**–**d** Analysis of SHH (**a**), PTCH1 (**b**), SMO (**c**), and GLI1 (**d**) expression levels in spinal cord biopsies of human healthy control (HC) and ALS patients; data are shown as standard box-and-whiskers of *n* = 10 HC and *n* = 10 ALS subjects; *p-value < 0.05 versus HC; two-sided Student’s *t*-test. **e** Interaction network of six selected genes (i.e. query) of SHH signaling pathway (in red) and the top-20 interactors genes (in white); interactions are shown as based on curated databases (red lines) or experimentally determined (black lines). **f** Enrichment KEGG analysis of genes showed in panel (**e**); significant pathways related to SHH signaling in humans are shown as −log of false discovery rate (FDR).

In order to evaluate the potential involvement of SHH signaling pathway in human ALS patients, we first performed a *z*-score analysis of a selected dataset containing mRNA expression data from spinal cord biopsies of *n* = 10 HC subjects and *n* = 10 ALS patients. Despite similar *SHH* mRNA expression levels (Fig. 7a), we found a significant reduction of *PTCH1* mRNA levels in the spinal cord of ALS patients (Fig. 7b). Notably, this reduction was not coupled with a reduction of *SMO* mRNA levels (Fig. 7c), but it was coupled with reduced mRNA levels of SHH-signaling pathway effector *GLI1* (Fig. 7d). This would suggest that SHH signaling pathway may represent a promising target for pharmacological treatment of MN diseases using SMO agonists.

Moreover, the interaction network of SHH signaling pathway genes *SHH*, *PTCH1*, *SMO*, *GLI1*, *GLI2,* and *GLI3* showed significant interactions, based on available databases or experimentally determined in humans, between the six selected genes (i.e. query) and the top-20 interactor genes (Fig. 7e). Enrichment KEGG analysis revealed significant modulation of pathways involved in chemokine signaling, axon guidance, synapses, and neurotransmission (Fig. 7f), thus supporting the idea that SHH modulation holds great relevance in human CNS homeostasis and compensatory processes.

## Discussion

Several studies have shown that alterations of spinal cord circuitries and MNs can start far earlier than the onset of symptoms [33, 34]. This suggests that compensatory changes occur in an attempt to maintain neuromuscular functions despite the progressive loss of motor units, and the possibility to target this plasticity coupled with efficient methods of early diagnosis would increase the chances of delaying the disease progression and hopefully promoting repair. Although genetic mouse models of ALS are necessary to model the human disease, reductionist animal models, such as the CTB-Sap model, are useful to dissect specific pathogenic mechanisms, since they are able to mimic a limited and controlled series of disease features. In our CTB-Sap model of spinal MN removal, the severity of the lesion and, as a consequence, the capability of self-restoration, can be modulated simply by choosing different doses of CTB-Sap. After administration of relatively low doses of toxin, mice were able to recover as soon as 4–6 weeks after the lesion [15]. Increased activity in the spared MNs and surrounding circuitries, likely driven by synaptic plasticity, could be responsible for these compensatory changes with possible involvement of SHH [35, 36].

In the present study, in order to test the effects of modulating the SHH pathway by the SMO agonist clobetasol, we used a dose of CTB-Sap able to ablate about 50% of resident MNs, and inducing a stable motor impairment, without any spontaneous amelioration up to 6 weeks post-lesion. The effects of clobetasol treatment were then evaluated from functional, metabolic, and pathophysiological aspects. After CTB-Sap injection, animals developed a rapid motor impairment of the affected hindlimb during the first week and these deficits appeared very stable during the entire experimental time-course in vehicle-treated animals. Interestingly, when animals were treated with clobetasol, a significant improvement of behavioral signs was observed as early as 3 weeks post-lesion and animals became able to near-normal walking.

These effects were coupled with reduced muscle denervation in clobetasol-treated mice. The post-mortem analysis of the spinal cord revealed a down-regulation of synaptophysin expression by the spared lumbar MNs, suggesting a decreased number of synapses on their somata after removal of neighboring MNs. Interestingly, lesioned animals treated with clobetasol showed an increased expression of synaptophysin, which is supposed to be linked to MN plasticity and functional restoration. Accordingly, the quantitative analysis of spinal cord metabolites has revealed a decrease of the inhibitory neurotransmitters GABA and glycine in lesioned animals. Moreover, although the level of glutamate was unchanged in the lumbar spinal cord after the lesion, we should consider that it acts onto a reduced population of MNs. Taken together, these findings suggest an attempt of spinal cord circuitries to compensate for the decreased neuromuscular activity, and the restoration of GABA and glycine levels after clobetasol treatment can suggest a rebalancing of neuronal activity promoted by the drug. Other critical aminoacids were affected by both MN removal and SMO modulation. Among them, we found significant alterations of lysine, tryptophan, arginine, and isoleucine, suggesting profound metabolic changes in the lesioned and treated spinal cord, involving glycolysis and tricarboxylic acid cycle. These changes are also present in patients, as well as in ALS experimental models [37, 38].

Our observation of astrocytes and microglial modulation is in line with increased levels of hypoxanthine and xanthine, the conversion of which produces a significant accumulation of reactive oxygen species. This could represent a major cause of neuronal suffering and it is also the rationale for edaravone treatment of ALS patients [39]. Notably, our results demonstrate that clobetasol is also able to reduce immune response not only modulating astrocytes and microglia polarization but also attenuating B and T NK lymphocyte infiltration.

The analysis of muscle also showed profound changes after CTB-Sap-induced denervation and as a result of treatment with clobetasol. Denervated GMs appeared atrophic but, interestingly, a five-fold increase of the number of CLN was detected in muscle sections, thus suggesting an attempt of regeneration [40]. The regenerative potential of the denervated muscle was boosted by administration of clobetasol, as demonstrated by the increased expression of Pax7 and by the increased cross-sectional area of muscle fibers, together with the reduction of CLN in comparison to the lesioned and vehicle-treated muscle. Taken together, the observed changes of GM morphology indicate that clobetasol may accelerate the muscle repairing processes.

The effect of CTB-Sap injection on muscle metabolism was observed in most of the metabolites under evaluation. A dramatic decrease in the ATP/ADP ratio, ECP, NAD^+^/NADH ratio, NADP^+^/NADPH ratio, concomitantly accompanied by an increase in oxypurines (hypoxanthine, xanthine, uric acid), IMP, nitrite, and nitrate, are clear indicators of mitochondrial dysfunction, also suggested by increased mitochondrial fission, with an imbalance in ATP production and consumption, consequent energy stress, the compensatory metabolic shift towards oxygen-independent glucose consumption through glycolysis, and oxidative/nitrosative stress with decreased antioxidant defenses. Among the various alterations of cell metabolism, those occurring to UDP-derivatives are certainly the most evident. The higher values of nucleotide sugars strongly suggest that the hexosamine biosynthetic pathway, representing the key process for the post-translational protein glycosylation, is highly active in damaged muscle. This finding may be indicative either of increased production of myokines or fibro/adipogenic processes, both being also reported as ALS-linked skeletal muscle changes [41, 42].

We hypothesize that clobetasol, acting as a glucocorticoid, could mobilize fatty acids and we found that, in denervated muscle, reduced malonil-CoA levels were present. Indeed, clobetasol was able to revert this phenomenon increasing fatty acid metabolism. Such results are in line with previously published reports linking hypolipidemia and dyslipidemia with ALS progression [43, 44].

Taken together, our data suggest that clobetasol exerts a pleiotropic effect in both the spinal cord and muscle finally resulting in neuromuscular plasticity. We can draw a number of conclusions from these observations. First, MNs depletion is associated with behavioral impairment and substantial neuromuscular alterations ranging from metabolic changes to the activation of immune response in the spinal cord, and from critical myopathy to energetic imbalance in the muscle. Second, the multitarget drug clobetasol acts by increasing Gli1 nuclear translocation in spinal astrocytes and spared MNs, reverting the known canonical SHH reduction that we observed in our experimental model of denervation [14, 36]. Third, robust inhibition of muscular fatty acid metabolism was observed in denervated mice and such a reduction was reverted by clobetasol.

In summary, these results confirm the existence of significant capacity of neuromuscular plasticity, which can be manipulated by drugs affecting a number of targets, including metabolism, reactive neuroglia and CNS infiltrating lymphocytes, synaptic plasticity, and muscle regeneration. Among these targets, particular attention should be paid to the SHH signaling, also in relation to MN diseases. The existence of an already approved drug-like clobetasol acting onto this pathway increases the interest in this promising potential therapeutic approach in ALS.

Interestingly, our work highlighting the role of SHH pathway in human ALS patients, suggests that the described results could be relevant for human ALS. Further studies also including SMO agonists in other animal models might provide additional evidence about the potential bench-to-bedside translation of the findings described here.

## Materials and methods

### Animal model

All experiments were performed in accordance with the principle of the Basel Declaration as well as to the European and Italian regulations (2010/63/EU and Italian D. Lgs. no. 26/2014). All efforts were made to replace, reduce and refine the use of laboratory animals. Moreover, the study was conducted in accordance with the recommendations of the local committee for animal welfare (OPBA, University of Catania, Catania, Italy); the protocol was approved by OPBA and by the Italian Ministry of Health. 48 male 129S1/SvImJ (Jackson Laboratory), 8–12 weeks old and weighing 25.6 ± 0.4 g were used. Animals were randomly assigned to different cages (*n* ≤ 5 animals per cage) and kept under constant temperature (23−25 °C) with ad libitum access to food and water. Mice were divided into three groups: HC group (no injection, *n* = 16), CTB-Sap clobetasol (injected with CTB-Sap and then treated with clobetasol propionate, *n* = 16), and CTB-Sap vehicle group (injected with CTB-Sap and then treated with drug vehicle only, *n* = 16). CTB-Sap injection was performed as previously described [45, 46]. Briefly, mice were anesthetized with isoflurane (4% for induction, 2% for maintenance) and then received two injections of the ribosome-inactivating and retrogradely transported toxin CTB-Sap (Advanced Targeting Systems, San Diego, CA, USA) into the medial and lateral left gastrocnemius with a toxin dose of 6 μg/2 μL in PBS per injection. Subgroups of CTB-Sap lesioned mice received an intraperitoneal injection of either clobetasol propionate (4 mg/kg) or vehicle alone, 7 days post lesion (dpl). Treatments were repeated once a week at 14, 21, 28, and 35 dpl.

Behavioral impairment was evaluated by two separate observers blind to the treatment analyzing the hind limb posture and gait capability, and assigning a clinical score in accordance with the following criteria described by Albano et al. [47, 48]. Animals were sacrificed at 42 dpl by intracardial perfusion and organs, i.e. spinal cords and GMs were dissected out, post-fixed, embedded in OCT or paraffin, respectively, and sectioned using a cryostat or a microtome.

### Open field grid walk test

Open field grid walk test for motor coordination impairment was performed at 7, 21, and 42 dpl using a “tracking camera” placed above the open field, to record and quantify the distance covered by every animal and a “counting camera”, placed in line with the grid, to quantify the number of footfalls within 2 min. For each performance, each animal was placed in a 40 × 40 cm grid (each mesh 1 cm^2^) and was free to move and explore during the behavioral test. The tracking and the counting videos were analyzed offline using Ctrax tracker software version 0.5.18 for Mac.

### Electromyography

EMG analysis was performed at 42 dpl using a portable two-channel EMG device (Myoquick, Micromed S.p.A., Mogliano Veneto, Treviso, Italy) with one bipolar concentric needle electrode inserted in the left GM and one ground electrode. Animals were anesthetized with isoflurane before the electromyographic recording. The spontaneous electrical activity of the muscle was recorded and then analyzed using System PLUS Evolution software by Micromed S.p.A. (Mogliano Veneto, Treviso, Italy).

### mRNA analysis

For quantification of mRNA levels of Dnm1l and actb in muscle, we used the QuantiGene Plex Magnetic Separation Assay kit (Affymetrix, Santa Clara, CA, USA) following the manufacturer’s instructions and as previously described [32]. The signal was detected with a Luminex instrument (Bio-Rad, Milan, Italy). For each sample, the average signal (MFI) for Dnm1l and actb were determined and, after average background signal subtraction, Dnm1l signal was normalized to the housekeeping gene signal.

### Immunohistochemistry

Rehydrated sections were subjected to a standard protocol of immunohistochemistry. Both muscle and spinal cord sections were subjected to a protocol of antigen retrieval (for AChE, Gfap and F4/80 staining) using an antigen retrieval buffer (0,1% Tween 20 in citrate buffer solution) and heating in microwave (5 min per 3 cycles). After samples were blocked with 3% H_2_O_2_ in PBS for 15 min at room temperature in a humidity chamber, they were washed in PBS and incubated for 40 min at room temperature in a humidity chamber with the following primary antibodies diluted in 0.3% Triton X100 in PBS: rat monoclonal anti-F4/80 (Bio-Rad, Cat#MCA497R, RRID: 323279, 1:100), rabbit monoclonal anti-AChE (Abcam, Cat#ab240274, RRID: AB_2857345,1:50), mouse monoclonal anti-GFAP (BD Biosciences, Cat#610566, RRID: AB_397916, 1:100). Then, samples were washed in 0.3% Triton in PBS three times for 5 min and incubated with pre-diluted biotinylated secondary antibody (Vector Laboratories, Burlingame, CA) and with R.T.U. VECTASTAIN Elite ABC Reagent (Vector Laboratories) for 30 min at room temperature in a humidity chamber, respectively. Samples were then washed in 0.3% Triton X100 in PBS for 5 min and exposed to a solution of 1% DAB, 0.3% H_2_O_2_ in PBS. Nuclei were counterstained with Mayer’s hematoxylin (Bio-Optica), dehydrated with increasing concentration of ethanol (70%, 95%, 100%) and xylene, and coverslipped with Entellan (Merck, Cat# 1.079.600.500).

For the analysis of GFAP by immunohistochemistry on the spinal cord, sections were counterstained with cresyl violet staining, dehydrated with the same procedure above and coverslipped with BrightMount (Aqueous Mounting Medium for Fluorescent Staining, Abcam, Cat#ab103746).

For neuronal or muscle fiber staining, spinal cord and muscle sections were stained with cresyl violet or hematoxylin/eosin, respectively.

For quantification of the number of GFAP positive cells, muscle fibers diameter distribution, CNL and F4/80 positive cells, *n* ≥ 3 equally spaced sections of the spinal cord or GM were analyzed and quantified by operators blind to the treatment using an Olympus BH2 microscope and Olympus CAST GRID software.

### Immunofluorescence

After antigen retrieval procedure as described above, sections were incubated with 10% NGS in PBS–0.3% Triton for 1 h at room temperature and then overnight at 4 °C with an appropriate combination of one of the following antibodies diluted in 1% NGS in PBS and 0.3% Triton. For spinal cord staining: rabbit polyclonal anti-Gli1 (Abcam, Cat#ab49314, RRID: AB_880198, 1:100); mouse monoclonal anti-NeuN (Millipore, Cat#MAB377, RRID: AB_2298772, 1:100); mouse monoclonal anti-GFAP (BD Biosciences, Cat# 610566, RRID: AB_397916, 1:500); goat polyclonal anti-AIF1/IBA1 (Novus, Cat# NB100-1028, RRID: AB_521594, 1:100); rabbit polyclonal anti-SYP (Santa Cruz Biotechnology, Cat#sc-9116, RRID: AB_2199007, 1:50); rabbit monoclonal anti-P-Stat3 (Cell signaling Technology, Cat# 9145, RRID: AB_2491009, 1:200). For muscle staining: mouse monoclonal anti-PAX7 (Abcam, Cat# ab199010, RRID: N.A., 1:250); rat monoclonal anti-F4/80 (Bio-Rad, Cat# MCA497R, RRID: AB_323279, 1:50); mouse monoclonal anti-iNOS (Santa Cruz Biotechnology, Cat# sc-7271, RRID: AB_627810, 1:50); rabbit polyclonal anti-ARG1 (Santa Cruz Biotechnology, Cat# sc-20150, RRID: AB_2958955, 1:50); mouse monoclonal anti-DRP1 (BD Biosciences, Cat# 611112, RRID: AB_398423, 1:100). Samples were then washed and incubated for 1 h at room temperature with appropriate secondary antibodies diluted 1:1000 in 1% NGS in PBS and 0.3% Triton: donkey anti-goat (Alexa Fluor 488, ThermoFisher Scientific, Cat#A-11055, RRID: AB_2534102 or Alexa Fluor 546, ThermoFisher Scientific, Cat#A-11056, RRID: AB_142628); goat polyclonal anti-rabbit (Alexa Fluor 488, Molecular Probes, Cat#A-11008, RRID: AB_143165; Alexa Fluor 546, Molecular Probes, Cat# A-11010, RRID: AB_2534077; Alexa Fluor 647, ThermoFisher Scientific, Cat#A-21244, RRID: AB_2535812); goat polyclonal anti-mouse (Alexa Fluor 488, ThermoFisher Scientific, Cat# A-11001, RRID: AB_2534069; Alexa Fluor 546, Molecular Probes, Cat#A-11003, RRID: AB_141370; Alexa Fluor 647, ThermoFisher Scientific, Cat# A-21235, RRID: AB_2535804); goat anti-rat (Alexa Fluor 488, Molecular Probes, Cat# A-11006, RRID: AB_141373). Nuclei were counterstained with DAPI 1:1000 in PBS. Slides were coverslipped with BrightMount (Abcam, Cat#ab103746).

For quantification of Gli1 fluorescence intensity (FI), Gli1 nuclear FI, iNOS FI, Arg1 FI, and mitochondrial length *n* ≥ 5 regions of interest per *n* ≥ 3 sections per animal were analyzed and quantified by operators blinded to the treatment using ImageJ v. 2.1.0/1.53c (Fiji) software.

### Flow cytometry

Flow cytometry analysis was performed on fresh tissues. Isolated spinal cords were kept in a cold IMDM medium supplemented with 5% FBS, 1% GlutaMAX, and 1% pen/strep and mechanically minced. Samples were then enzymatically digested at 37 °C for 30 min with a cocktail of 2 mg/ml collagenase IV, 0.2 mg/ml dispase, and 0.1 mg/ml DNase I in supplemented IMDM. Then, the cell suspension was homogenized by passaging through 40 µm cell strainers using 10 ml of supplemented IMDM for sample. For myelin removal, the suspension was mixed with 90% isotonic Percoll diluted in PBS 10× and then centrifuged for 20 min at 800×*g*. Cell pellet was resuspended in cold buffer (MACS BSA diluted in autoMACS rinsing solution) and centrifuged twice at 4 °C for 5 min at 300*g*, resuspending pellet with cold buffer and flow cytometry staining buffer respectively, obtaining 1,000,000 cells/100 µl/sample. Samples were finally incubated for 15 min at 4 °C with the following monoclonal antibodies: anti-mouse CD45 (Miltenyi Biotech, Cat#130-110-802, RRID: AB_2658222), CD11b (Miltenyi Biotech, Cat#130-113-803, RRID: AB_2819369), F4/80 (Biolegend, Cat#123118, RRID: AB_893477), Ly-6G (Miltenyi Biotech, Cat#130-117-500, RRID: AB_2727967), CCR2 (Miltenyi Biotech, Cat#130-117-548, RRID: AB_2727981), CX3CR1 (Biolegend, Cat#149006, RRID: AB_2564315), CD206 (Biolegend, Cat#141717, RRID: AB_2562232), and CD80 (Miltenyi Biotech, Cat#130-116-462, RRID: AB_2727559) antibodies.

In all experiments, viobility fixable dye (Miltenyi Biotech, Cat#130-109-816) was used to label dead cells. Data were collected on a MACSQuant Analyzer (Miltenyi Biotech) and analyzed using Flowlogic software (Miltenyi Biotech).

Data analysis was performed as described elsewhere [49, 50]. Briefly, it was based on a multi-step bioinformatics approach: (1) reading FCS data; (2) building a self-organizing map (SOM) for clustering; (3) performing a dimensionality reduction through principal component analysis (PCA) and uniform manifold approximation and projection (UMAP); (4) perform a supervised identification of each cluster; (5) performing a statistical analysis on the abundance of each population according to their origin (ANOVA). Besides the characterization of the myeloid compartment, the unsupervised clustering algorithm identified two different lymphocytes clusters: one with strong positivity for HLA-DR, that we identified as B cells and a second one that includes residual T and NK cells (T/NK) which cannot be separated with the current markers panel.

### Tissue preparation and HPLC analysis of metabolites

All animals of the three groups (controls, CTB-Sap vehicle, and CTB-Sap clobetasol) underwent the same surgical procedure to remove spinal cord and GMs. Once removed, tissues were immediately immersed in liquid nitrogen, weighed and deproteinized by homogenization in ice-cold HPLC-grade CH_3_CN + 10 mM KH_2_PO_4_ pH 7.40 (3:1; v-v), using an Ultra-Turrax (Janke and Kunkel, Staufen, Germany) at 24,000 rpm/min for 90 s [51–55]. Following centrifugation at 20,690×*g* for 10 min at 4 °C, clear supernatants were supplemented with HPLC-grade chloroform, vigorously agitated and centrifuged for 5 min in a top-bench centrifuge at the maximal speed. The upper aqueous phase was used for the HPLC analyses of metabolites. The simultaneous separation and quantification of high-energy phosphates, oxidized and reduced nicotinic coenzymes, purines, pyrimidines, antioxidants, oxidative/nitrosative stress biomarkers and N-acetylaspartate (NAA) was performed as described in detail elsewhere. Amino acids (AA) and amino group-containing compounds (AGCC) were separated and quantified according to chromatographic conditions previously set up in our laboratory. In both cases, the HPLC apparatus consisted of a Spectra System P4000 pump, connected to a Hypersil C-18 250 × 4.6 mm, 5 µm particle size column (provided with its own guard column) and to a highly sensitive UV6000 LP diode array detector equipped with a 5 cm light path flow cell (ThermoFisher Scientific, Rodano, Milano, Italy). Overall, the two analyses allowed measuring the concentrations of 61 water-soluble low molecular weight compounds in tissue extracts, thanks to the comparisons with appropriate ultrapure standard mixtures with known concentrations.

### Principal components analysis

Principal components analysis (PCA) was performed on spinal cord and GMs metabolites data. Explained variances and the quality of representation of the variables on the factor map expressed as square cosine (Cos2) are shown. PCA is expressed as a biplot of variables and key-colored arrows representing each variable are also shown. Analyses were carried out using the RStudio software (Version 1.0.153, for Mac), fitted with corr plot, FactoMineR, and Factoextra packages for computing and visualizing PCA diagrams.

### Human gene expression

For human ALS data, we used the NCBI Gene Expression Omnibus (GEO) database (http://www.ncbi.nlm.nih.gov/geo/) to select *n* = 10 healthy and *n* = 10 ALS patients cervical spinal cord transcriptome dataset (GSE26927) analyzing the expression levels of *n* = 6 selected genes (i.e. PTCH1, SMO, SHH, GLI2, GLI1, and GLI3). Mesh terms “Spinal Cord”, “ALS” and “Human” were used to identify potential datasets of interest. The dataset selected was downloaded and analyzed using MultiExperiment Viewer (MeV) software. Statistical analysis was performed using GraphPad Prism software version 5.00 for Mac and MeV software. Interaction network of SHH signaling pathway was obtained using String (https://string-db.org/) online tool for gene ontology and KEGG enrichment analysis.

### Quantification and statistical analysis

The sample size for each experiment is reported in figure legends. No statistical methods were used to predetermine sample sizes, but our sample sizes were similar to those reported in previous publications [15, 18, 48, 56]. Mice were randomly assigned to experimental groups. No data points or animals were excluded from the analysis. For statistical analyses, a two-tailed unpaired Student’s *t*-test was used for comparison of *n* = 2 groups. For comparison of *n* ≥ 3 groups, one-way or two-way analysis of variance (ANOVA), or repeated-measures ANOVA with Holm–Sidak post-hoc test for multiple comparisons were used where appropriate. Data are presented as the mean ± standard error of the mean (SEM) unless otherwise stated. Data analysis was performed using GraphPad Prism software version 5.00 or RStudio software version 1.0.153. A value of *p* < 0.05 was considered statistically significant and symbols used to indicate statistical differences are described in figure legends.

## Supplementary information

Supplementary Figures

## Data Availability

The datasets used and/or analyzed during the current study are available from the corresponding author on reasonable request.
