# Supplementary material for: Clobetasol promotes neuromuscular plasticity in mice after motoneuronal loss via sonic hedgehog signaling, immunomodulation and metabolic rebalancing
Source: Cell Death Dis. 2021 Jun 16;12(7):625. doi: 10.1038/s41419-021-03907-1 (PMC8209072; doi:10.1038/s41419-021-03907-1)
Supplement: Supplementary file 1 — Supplementary Figures [file 41419_2021_3907_MOESM1_ESM.pdf]

Supplementary figures and figure legends for:

Clobetasol promotes neuromuscular plasticity in mice after motoneuronal loss via sonic hedgehog signaling, immunomodulation and metabolic rebalancing

Nunzio Vicario, Federica M. Spitale, Daniele Tibullo, Cesarina Giallongo, Angela M. Amorini, Grazia Scandura, Graziana Spoto, Miriam W. Saab, Simona D'Aprile, Cristiana Alberghina, Renata Mangione, Joshua D. Bernstock, Cirino Botta, Massimo Gulisano, Emanuele Buratti, Giampiero Leanza, Robert Zorec, Michele Vecchio, Michelino Di Rosa, Giovanni Li Volti, Giuseppe Lazzarino, Rosalba Parenti, Rosario Gulino.

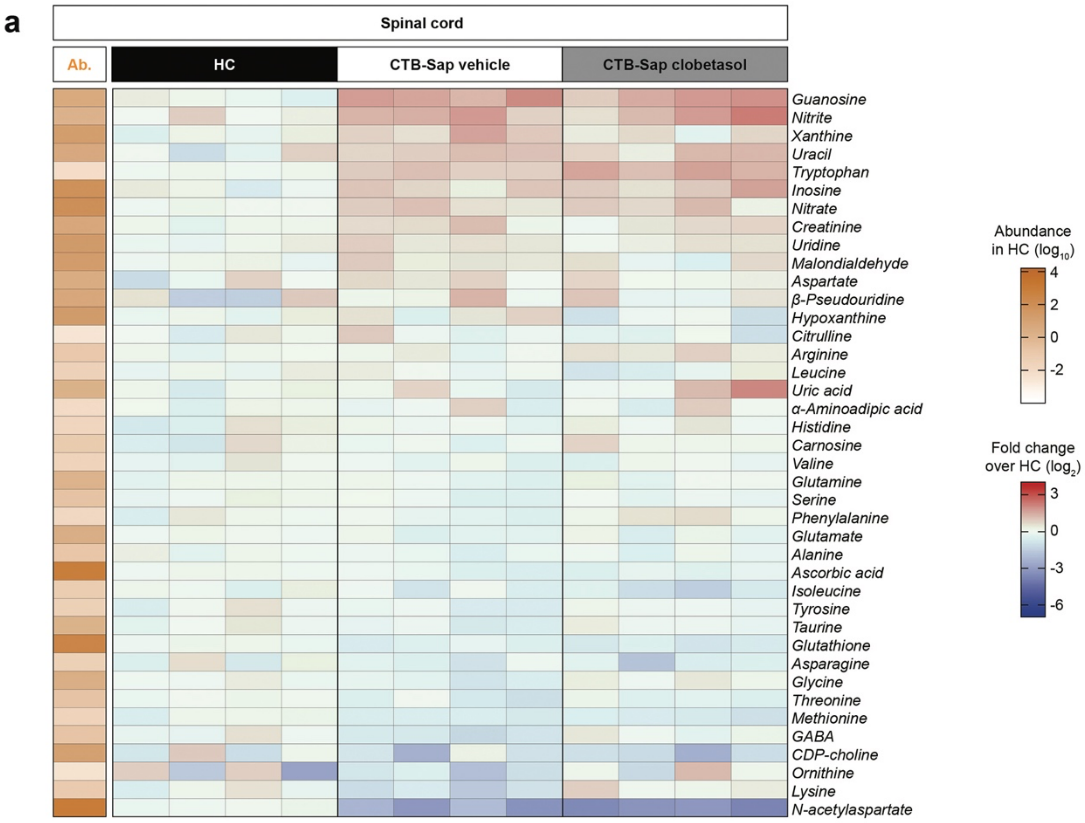

Fig. S1 l Clobetasol modifies metabolism of MN-depleted spinal cord. a, Heat maps of 40 metabolites in HC, CTB-Sap vehicle and CTB-Sap clobetasol spinal cord at 42 dpl showing the abundance (Ab.) in HC and the relative changes in CTB-Sap vehicle and CTB-Sap clobetasol as compared to HC; data are shown $\log_{10}$ abundance and $\log_2$ FC over HC of n = 4 mice per group.

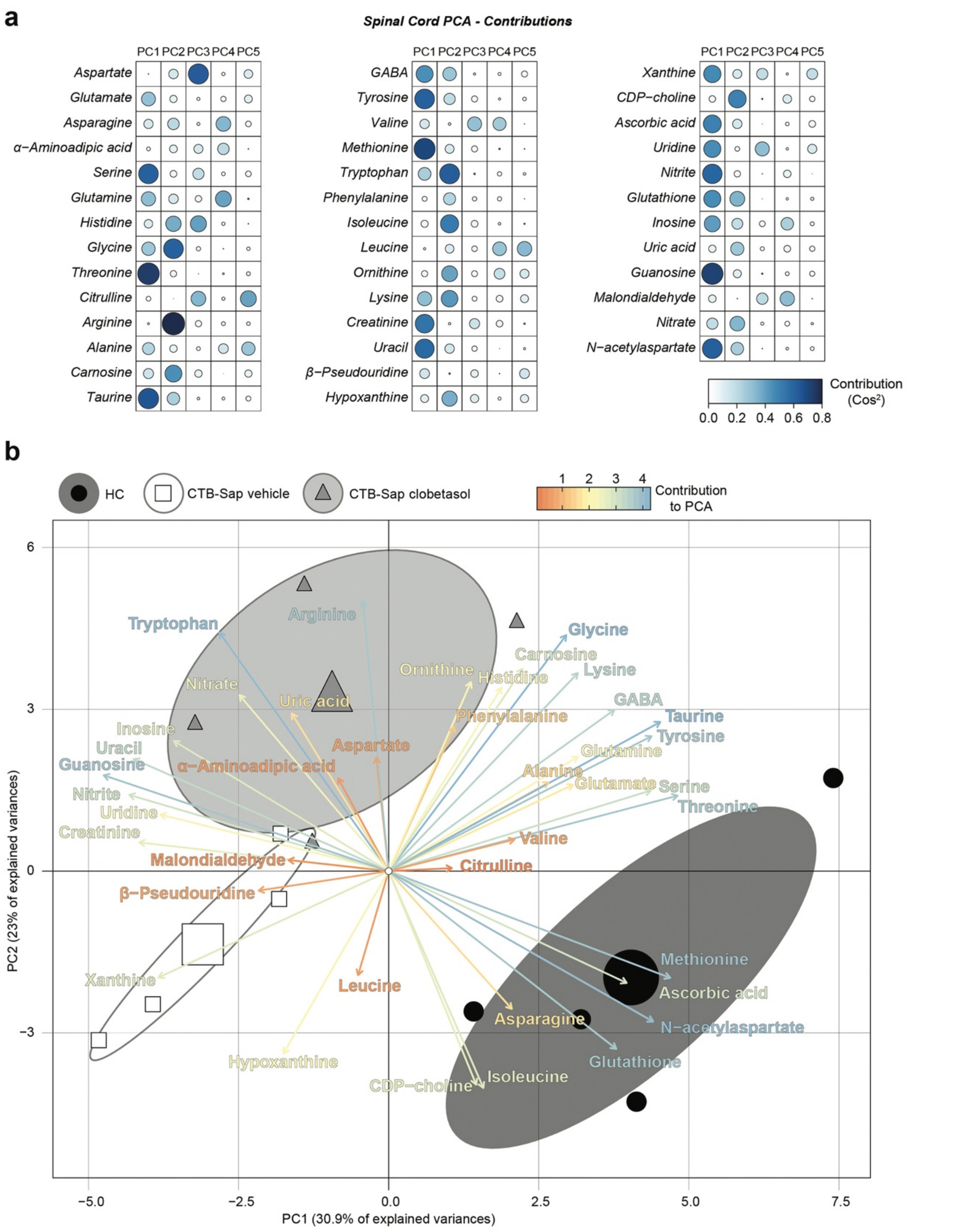

Fig. S2 I Principal components analysis (PCA) of spinal cord metabolites in HC, CTB-Sap vehicle and CTB-Sap clobetasol mice. a, Factor map of quality plot representation of the variables on PC1-PC5 expressed as square cosine (Cos²). **b**, PCA biplot of 40 metabolites for HC, CTB-Sap vehicle and CTB-Sap clobetasol; key-colored arrows represent the contribution of each variable to the PCA; small circles/squares/triangles represent single subjects and large circles/squares/triangles represents the mean point of the confidence ellipses.

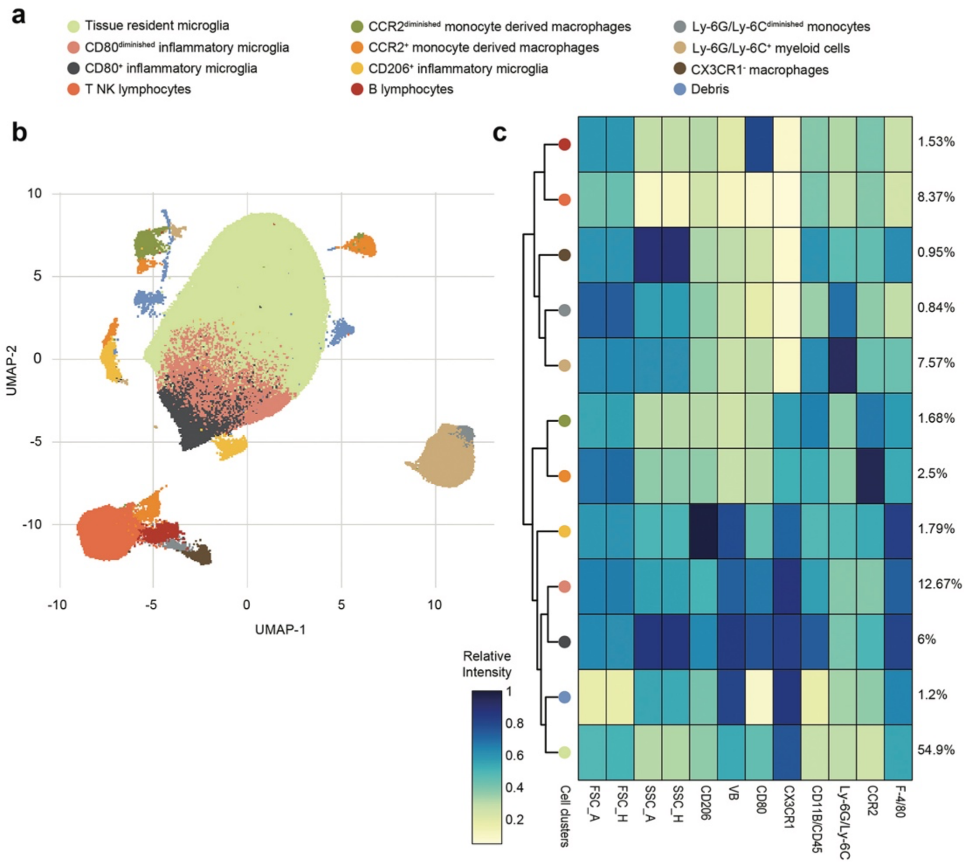

Fig. S3 I Cell populations identified by multi-step bioinformatics analysis of flow cytometry panel. a, b, Uniform Manifold Approximation and Projection (UMAP) representation of the populations identified through flow-cytometry by “fusing” all the events from the 12 samples (4 mice for 3 conditions).

c, Heatmap showing the expression of each marker in each population. The differential expression was used to assign, in a supervised manner, each cluster to each immune population. The overall abundance of each subpopulation is reported within the heatmap.

a

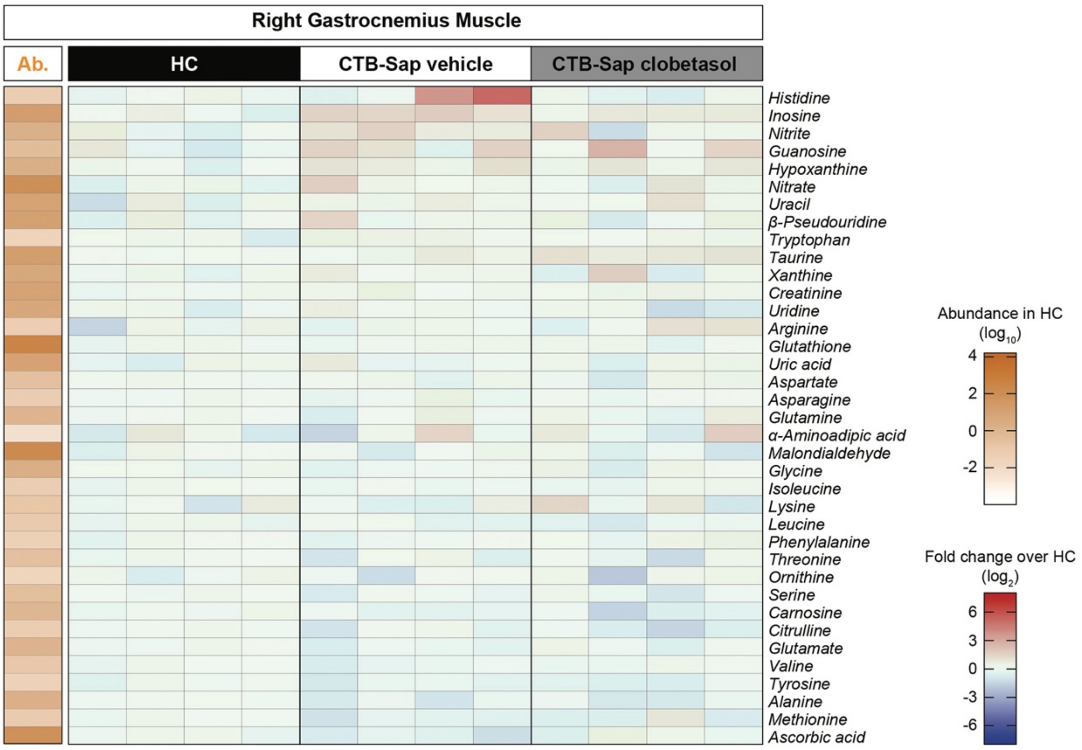

b

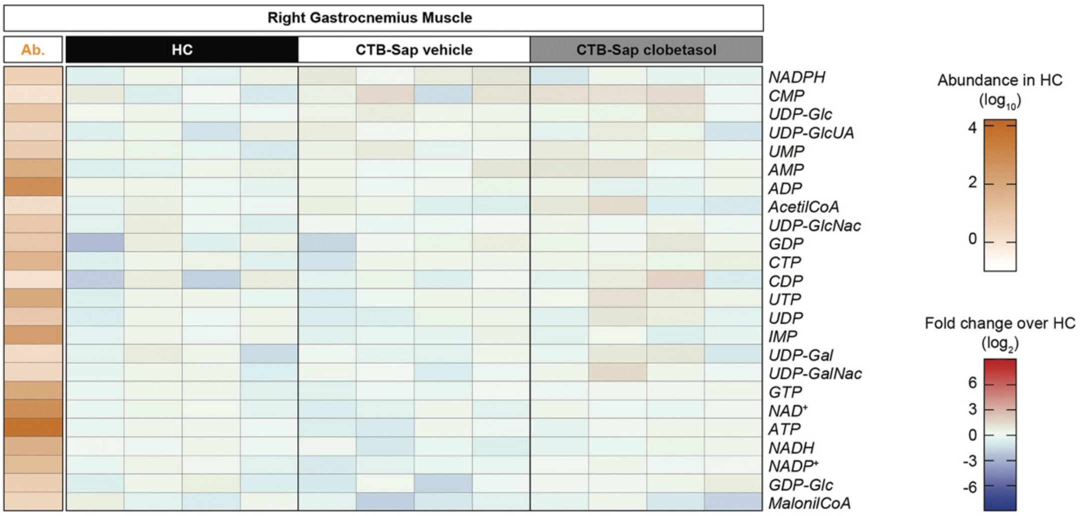

Fig. S4 l Effects of clobetasol onto contralateral, unaffected muscle. a, Heat maps of 37 metabolites in the right GM HC, CTB-Sap vehicle and CTB-Sap clobetasol at 42 dpl showing the abundance (Ab.) in HC and the relative changes in CTB-Sap vehicle and CTB-Sap clobetasol as compared to HC; data are shown as log_{10} abundance and log_2 FC over HC of n = 4 mice per group.

b, Heat maps of 24 metabolites in the right GM HC, CTB-Sap vehicle and CTB-Sap clobetasol at 42 dpl showing the abundance (Ab.) in HC and the relative changes in CTB-Sap vehicle and CTB-Sap clobetasol as compared to HC; data are shown as log_{10} abundance and log_2 FC over HC of n = 4 mice per group.

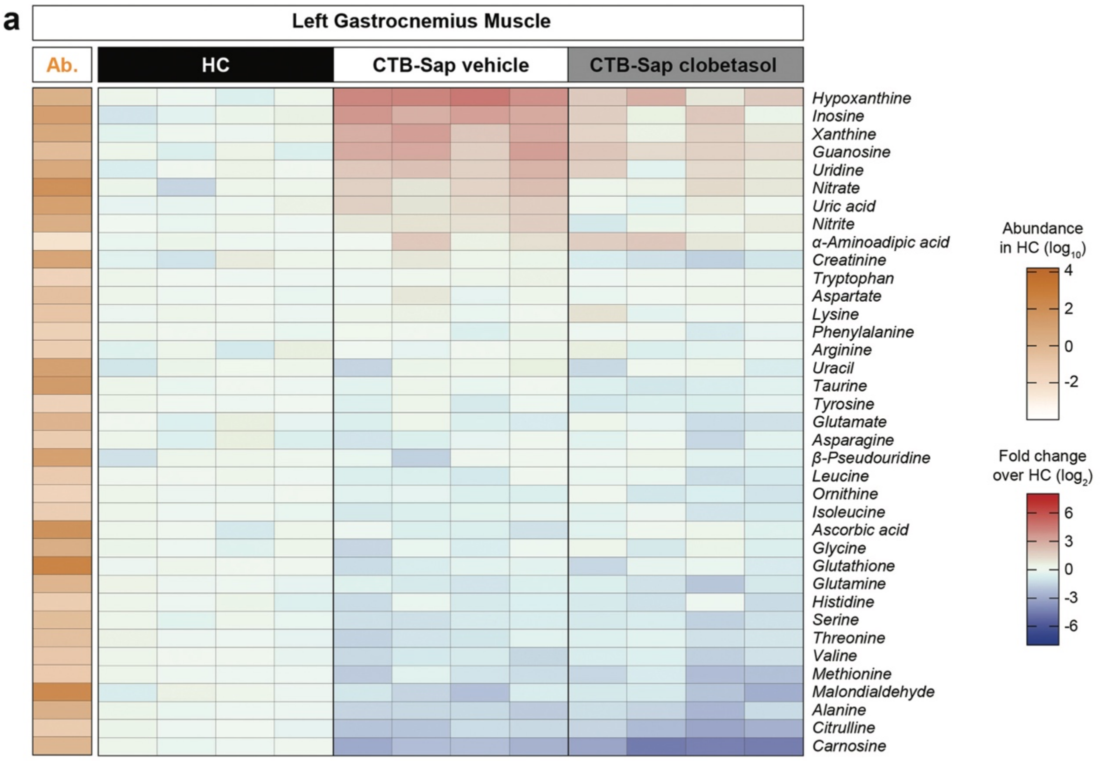

Fig. S5. Clobetasol modifies metabolism of denervated muscle. a, Heat maps of 37 metabolites in the left GM HC, CTB-Sap vehicle and CTB-Sap clobetasol at 42 dpl showing the abundance (Ab.) in HC and the relative changes in CTB-Sap vehicle and CTB-Sap clobetasol as compared to HC; data are shown as log_{10} abundance and log_2 FC over HC of n = 4 mice per group.

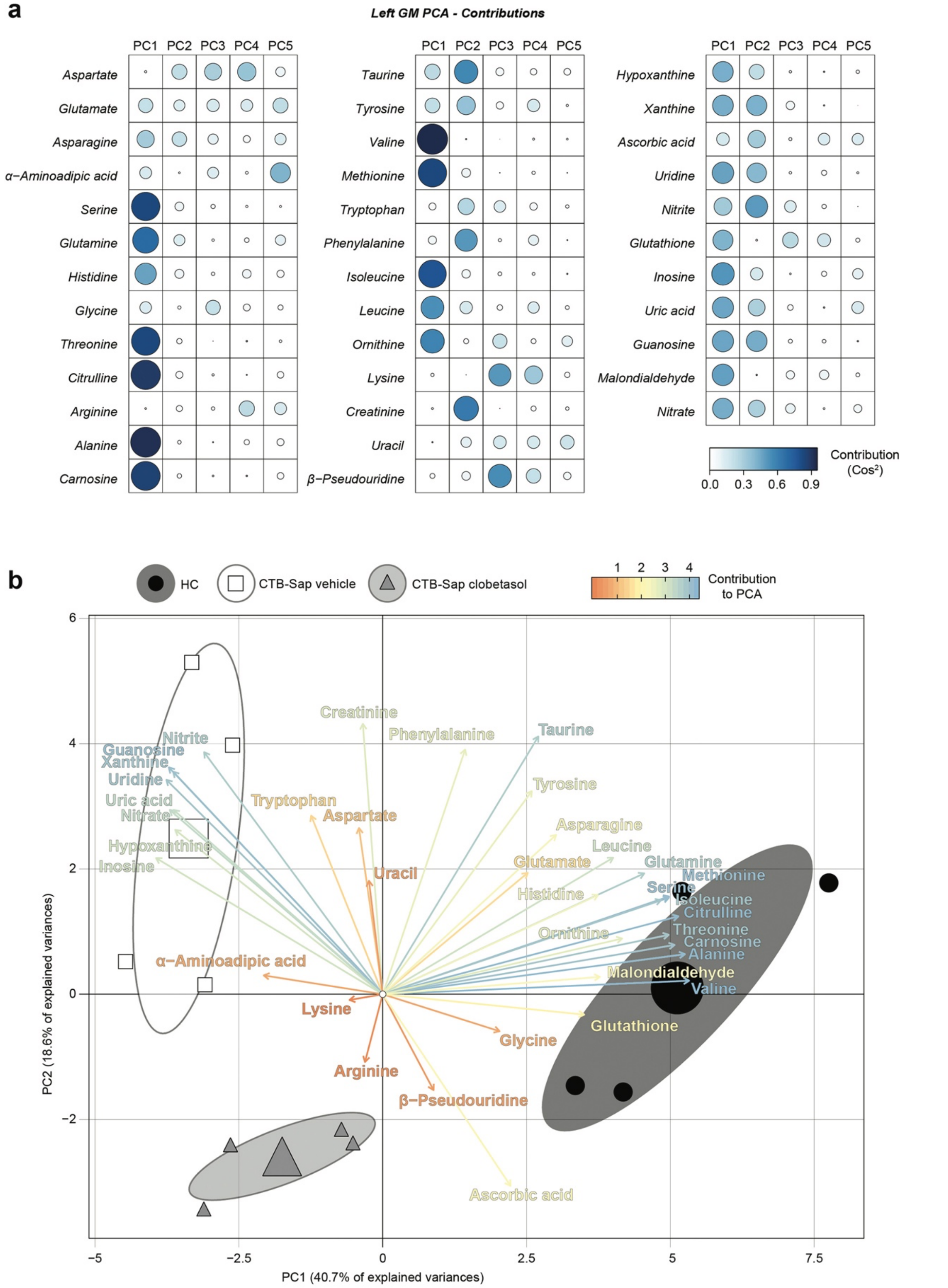

Fig. S6. Principal components analysis (PCA) of left GM metabolites of HC, CTB-Sap vehicle and CTB-Sap clobetasol mice. a, Factor map of quality plot representation of the variables on PC1-PC5 expressed as square cosine (Cos²). **b**, PCA biplot of 37 metabolites for HC, CTB-Sap vehicle and CTB-Sap clobetasol; key-colored arrows represent the contribution of each variable to the PCA; small circles/squares/triangles represent single subjects and large circles/squares/triangles represents the mean point of the confidence ellipses.

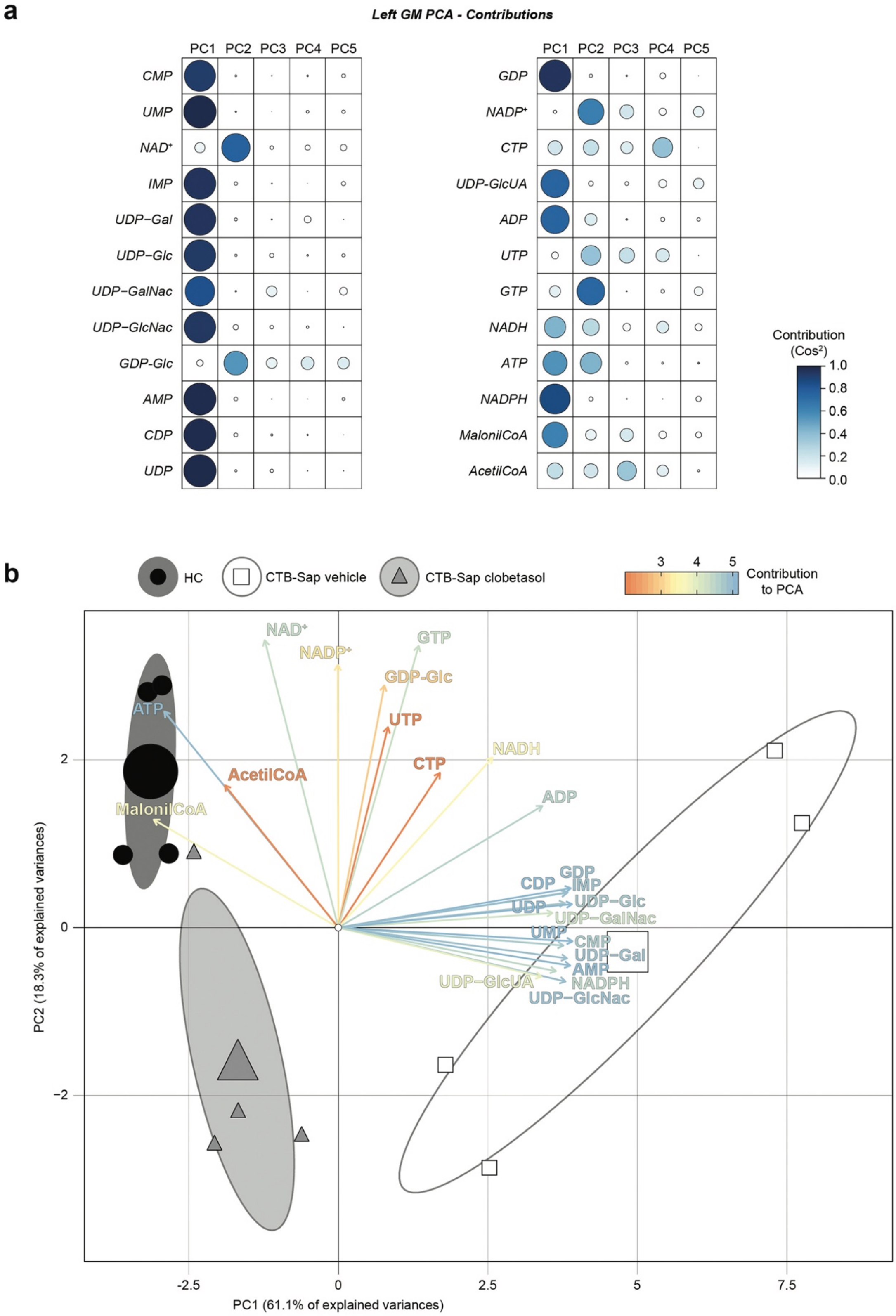

Fig. S7. Principal components analysis (PCA) of left GMs metabolites of HC, CTB-Sap vehicle and CTB-Sap clobetasol mice. a, Factor map of quality plot representation of the variables on PC1-PC5 expressed as square cosine (Cos^2). **b,** PCA biplot of 24 metabolites for HC, CTB-Sap vehicle and CTB-Sap clobetasol; key-colored arrows represent the contribution of each variable to the PCA; small circles/squares/triangles represent single subjects and large circles/squares/triangles represents the mean point of the confidence ellipses.
